# Baseline Susceptibility of the Field Populations of *Ostrinia furnacalis* in Indonesia to the Proteins Cry1A.105 and Cry2Ab2 of *Bacillus thuringiensis*

**DOI:** 10.3390/toxins15100602

**Published:** 2023-10-07

**Authors:** Y. Andi Trisyono, Valentina E. F. Aryuwandari, Teguh Rahayu, Samuel Martinelli, Graham P. Head, Srinivas Parimi, Luis R. Camacho

**Affiliations:** 1 Department of Plant Protection, Faculty of Agriculture, Universitas Gadjah Mada, Yogyakarta 55281, Indonesia; 2 Regulatory Science, Bayer Crop Science US, Chesterfield, MO 63017, USA; 3 Bayer Crop Science Ltd., Hyderabad 500081, India; 4 Bayer (South East Asia) Pte Ltd., 2 Tanjong Katong Road #07-01, Paya Lebar Quarter 3, Singapore 437161, Singapore

**Keywords:** Asian corn borer, Cry1A.105, Cry2Ab2, Indonesia, resistance monitoring, susceptibility

## Abstract

Genetically modified MON 89034 corn (*Zea mays* L.) expressing *Bacillus thuringiensis* (*Bt*) insecticidal proteins, viz. Cry1A.105 and Cry2Ab2, is a biotechnological option being considered for the management of the major corn pest in Indonesia, the Asian corn borer (*Ostrinia furnacalis* (Guenée) (Lepidoptera: Crambidae)). As a part of a proactive resistance-management program for MON 89034 corn in Indonesia, we assessed the baseline susceptibility of field-collected populations of *O. furnacalis* to Cry1A.105 and Cry2Ab2 proteins. Dose–response bioassays using the diet-dipping method indicated that the lethal concentration (LC_50_) values of Cry1A.105 and Cry2Ab2 in 24 different field populations of *O. furnacalis* ranged from 0.006 to 0.401 µg/mL and from 0.044 to 4.490 µg/mL, respectively, while the LC_95_ values ranged from 0.069 to 15.233 µg/mL for Cry1A.105 and from 3.320 to 277.584 µg/mL for Cry2Ab2. The relative resistance ratios comparing the most tolerant field populations and an unselected laboratory population were 6.0 for Cry1A.105 and 2.0 for Cry2Ab2 based on their LC_50_ values. Some field populations were more susceptible to both proteins than the unselected laboratory population. The LC_99_ and its 95% fiducial limits across the field populations were calculated and proposed as candidate diagnostic concentrations. These data provide a basis for resistance monitoring in *Bt* Corn and further support building resistance-management strategies in Indonesia.

## 1. Introduction

The Asian corn borer, *Ostrinia furnacalis* (Guenée), is one of the major pests of corn (*Zea mays* Linnaeus) in Southeast Asia, including the Indonesian archipelago, Vietnam, the Philippines, and China [1]. Areekul [2] and Camarao [3] reported two generations of *O. furnacalis* infestation in each growing season in tropical areas with 24–30 total development days for each generation. Damage from *O. furnacalis* occurs not only in the stem of corn plants but also in the whorl, tassel, and ears [4,5]. In vegetative stage plants, the newly hatched larvae feed on young leaf whorls, resulting in holes in the leaves that can widen as the larvae grow and feed more vigorously. In the reproductive stage, the larvae feed on tassel, then bore into the stem, making tunnels, and might continue to feed on the ears [2]. Da-Lopez et al. [6] reported that the presence of *O. furnacalis* egg masses was as high as nine egg masses per plant in fields in Sleman, Yogyakarta. A recent study (2018–2019) in Lampung and Central Java showed that *O. furnacalis* infestations were present in as many as 95% of corn plants with an average of four holes per stalk and gallery lengths of 4–6 cm [7]. This level of damage was above the economic threshold for this insect, estimated as one larva (hole) per stalk [8,9]. One *O. furnacalis* larva boring per plant during V10, R1, or R2 resulted in grain yield losses of 4.94%, 4.56%, or 3.76%, respectively [9].

The economic damage on corn indicates the need for effective and ecologically sound management practices for *O. furnacalis* in Indonesia and other countries in Southeast Asia [10,11], where growers have similar challenges due to this species attacking corn plants [5,12,13]. Chemical control using insecticides is the most commonly practiced control measure in Indonesia for the management of *O. furnacalis* [7]. Genetically modified corn plants expressing *Bacillus thuringiensis* (*Bt*) insecticidal proteins have been commercialized and are widely grown in corn-growing countries, such as the United States, Brazil, Argentina, Canada, South Africa, the Philippines, and Vietnam for the successful management of corn stalk borers, corn ear feeders, and fall armyworm (*Spodoptera frugiperda* (J.E. Smith)) [14,15,16]. Several *Bt* corn technologies are currently in the process of registration in Indonesia [17], including the MON 89034 corn. MON 89034 is a pyramided transgenic corn event expressing two *Bt* proteins, Cry1A.105 and Cry2Ab2, that are highly effective against key lepidopteran corn pests [18,19,20]. MON 89034 has been approved for commercialization in the Philippines since 2010 and in Vietnam since 2015 as a viable alternative for the sustainable control of *O. furnacalis* and other corn lepidopteran pests [15,21,22]. In September 2021, MON 89034 was approved as a registered product to control *S. frugiperda* in the Philippines [23].

The development of resistance in populations of the target pests poses the risk to the sustainability of *Bt* crops [24,25,26]. In Indonesia, the risk of resistance development could be higher due to the year-round cultivation of corn which may result in continuous pressure of selection once MON 89304 is commercialized. The planting of refuges and the adoption of technologies expressing multiple *Bt* proteins with an independent mode of action significantly reduces the risk of resistance development in populations of the target pests [27,28]. Because MON 89034 corn expresses two *Bt* proteins of different mechanisms of action targeting *O. furnacalis*, its inherent resistance risk is likely lower than that for single-gene *Bt* plants [29]. Nevertheless, it is important to monitor the susceptibility of *O. furnacalis* populations to Cry1A.105 and Cry2Ab2 proteins after the introduction of MON 89034 in Indonesia. Establishing the baseline susceptibility of *O. furnacalis* to Cry1A.105 and Cry2Ab2 is important for resistance-monitoring programs in Indonesia. The assessment of the baseline susceptibility would not only allow the assessment of the natural variation among field populations but can also be used to document shifts in susceptibility likely resulting from selection for resistance [30,31]. We hypothesize that there will be differences in the sensitivity of *O. furnacalis* populations. The goals of this study were to establish the baseline susceptibility of *O. furnacalis* to Cry1A.105 and Cry2Ab2 proteins in Indonesia and to estimate Cry1A.105 and Cry2Ab2 concentrations to be used as candidate diagnostic concentrations to monitor the development of resistance in *O. furnacalis* in Indonesia.

## 2. Results

### 2.1. Susceptibility of O. furnacalis to Cry1A.105

Populations of *O. furnacalis* collected from six provinces in 2013–2015 showed variation in susceptibility to Cry1A.105 (Table 1). The LC_50_ values for the field-collected populations ranged from 0.006 to 0.401 µg/mL, and for the laboratory population, these were 0.067 µg/mL. The LC_95_ of the laboratory population was 0.890 µg/mL, while those of the field-collected populations varied from 0.069 to 15.233 µg/mL. The resistance ratios (RRs) of the 24 field populations relative to the laboratory population were 0.1–6.0 based on the LC_50_ values (Table 1). Some field populations were more susceptible (RR < 1) to the protein than the laboratory population, while other field populations were more resistant (RR > 1) than the laboratory population. The difference between LC_50_ values of any two populations from Cry1A.105 assays was considered significant if their 95% fiducial limits (FLs) did not overlap [32,33].

### 2.2. Susceptibility of O. furnacalis to Cry2Ab2

Populations of *O. furnacalis* collected from six provinces in 2013–2015 also showed variation in susceptibility to Cry2Ab2, as evidenced by the 0.1–2.0 RR values based on the LC_50_ (Table 2). The LC_50_ values of Cry2Ab2 against 24 field-collected populations ranged from 0.044 µg/mL to 4.490 µg/mL, whereas that of the laboratory population was 2.276 µg/mL. The LC_95_ value of the laboratory population was 22.984 µg/mL, and for the 24 field populations, it ranged from 3.320 to 277.584 µg/mL. Similarly to responses observed in the Cry1A.105 assays, some field populations were more susceptible to Cry2Ab2 than the laboratory population. The difference between LC_50_ values of any two populations from Cry2Ab2 assays was considered significant if their 95% fiducial limits (FLs) did not overlap [32,33].

### 2.3. Candidate Diagnostic Concentrations

The *O. furnacalis* LC_99_ value for Cry1A.105 was significantly higher than its LC_95_ value as indicated by non-overlapping lower and upper limits of the 95% FL (Table 3). However, there was no significant difference observed between LC_99_ and LC_95_ values for Cry2Ab2. We propose the LC_99_ and its upper and lower fiducial limits (95% FLs) as the candidate diagnostic concentrations: 13.240 (6.716–33.831) µg/mL for Cry1A.105 and 127.320 (46.616–676.792) µg/mL for Cry2Ab2. These concentrations need to be tested with several field populations for further validation, followed by the selection of one diagnostic concentration for each protein to be used in monitoring programs.

## 3. Discussion

There is a need to develop a robust insect resistance-management (IRM) strategy for MON 89034 in Indonesia to prolong its durability in the field and, thus, delay the development of practical resistance once the event is approved for cultivation [27,34,35]. Baseline susceptibility data are essential for resistance-monitoring purposes, particularly to provide information on the level of susceptibility of *O. furnacalis* to Cry1A.105 and Cry2Ab2 before the introduction of MON 89034 corn and to provide benchmark data for future comparison to detect susceptibility shifts. In this study, the baseline susceptibility of *O. furnacalis* to Cry1A.105 and Cry2Ab2 was established based on the populations collected from major corn-producing provinces in Indonesia.

Differences in susceptibility to *Bt* proteins before the onset of resistance have been reported among geographically distinct populations in many different species of insects attacking corn [20,36,37,38]. For *O. furnacalis*, several studies have been conducted using five proteins, i.e., Cry1Ab, Cry1Ac, Cry1F, Cry1A.105, and Cry2Ab2. Field-collected populations of *O. furnacalis* in Vietnam differed in susceptibility to Cry1Ab by up to three-fold, which was reflective of natural variability among the 11 populations used in the study [39]. In China, the differences in susceptibility for this species were up to eight-fold for Cry1Ab [40], and in different study, up to two-fold for Cry1Ab, Cry1Ac, and Cry1F [41]. Contrastingly, in yet another study that assayed the bioactivity of Cry1Ab with 25 field populations of *O. furnacalis* in China [33], 80-fold and 309-fold variations at LC_50_ and LC_95_, respectively, were demonstrated. Furthermore, Alcantara et al. [20] reported that the differences in the Philippines were six- and seven-fold to Cry1A.105 and Cry2Ab2, respectively.

We reported higher levels of variation in the susceptibility of *O. furnacalis* field populations to Cry1A.105 and Cry2Ab2 than were reported in the Philippines. However, direct comparison between these two studies (diet dipping vs. diet overlay) was not possible since different bioassay procedures were employed. Differences in the susceptibility of *O. furnacalis* to these two proteins may represent natural variation among populations because transgenic corn has not yet been planted commercially in Indonesia, and commercial *Bt* formulations for controlling *O. furnacalis* are not commonly utilized by Indonesian farmers [4]. A high difference in the susceptibility of field-collected populations was previously reported with *O. furnacalis* to Cry1Ab [33] and in *Helicoverpa armigera* to Cry1Ac (67-fold) in India [42]. In addition, the innate heterogeneity within insect populations tested in routine bioassays using the same methodology may account for three- to six-fold, or even twelve-fold, variation in laboratory-reared population comparisons [43,44]. Therefore, the heterogeneity may be even greater across field-derived populations, and the method chosen for bioassay needs careful consideration in the context of resistance monitoring.

Cry1A.105 was more toxic than Cry2Ab2 against *O. furnacalis* in Indonesia (Table 1 and Table 2) and in the Philippines [20]. Similar results were reported for one species of corn stem borer, *Chilo partellus*, in India [38]. In contrast, Cry2Ab2 was more toxic than Cry1A.105 against the other species of corn borer in India, *Sesamia inferens* [38]. The same researchers also reported that Cry1A.105 was more toxic than Cry2Ab2 against *H. armigera*. Hernandez-Rodriguez et al. [45] also reported that Cry1A.105 was more toxic than Cry2Ab2 to *Ostrinia nubilalis* and *S. frugiperda*. These studies provide evidence that different species of corn borers may have different levels of susceptibility to different proteins and that their susceptibility may differ among geographically distinct populations.

A laboratory concentration of Cry1A.105 and Cry2Ab2 that reliably causes 99% mortality provides a candidate for use as a discriminating concentration [46]. The proposed diagnostic concentrations of 6.7, 13.2, and 33.8 µg/mL for Cry1A.105 and of 46.6, 127.3, and 676.8 µg/mL for Cry2Ab2 need to be validated against field-collected populations. Based on these tests, the determination of diagnostic concentrations for each protein is planned and these should be available before the commercialization of MON 89304.

The expression of Cry1A.105 and Cry2Ab2 in MON 89034 corn varies depending on the tissues with the highest expression occurred in the young leaves of V2–V4 with the average of 520 and 180 µg/g dry weight tissue [47]. This information in combination with baseline data may be used in developing IRM strategies in Indonesia.

## 4. Conclusions

This study reports the baseline susceptibility of distinct geographical *O. furnacalis* populations of Indonesia to Cry1A.105 and Cry2Ab2 proteins. Our findings demonstrate that *O. furnacalis* populations are highly sensitive to Cry1A.105 and Cry2Ab2 proteins, although there are differences in sensitivity among populations, which is a natural variation. The baseline susceptibility data are used to establish candidate diagnostic concentrations for further validations. The data on baseline susceptibility and the diagnostic concentrations provide valuable tools for future resistance-monitoring programs to detect early shifts in sensitivity among the field populations of *O. furnacalis* once transgenic corn expressing these two proteins is commercialized in Indonesia.

## 5. Materials and Methods

### 5.1. Field-Collected Populations

**Figure 1:**
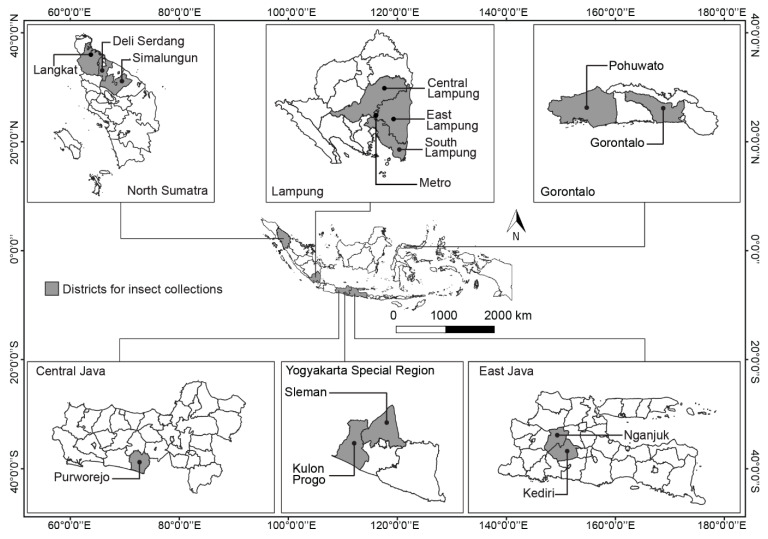
The six provinces in Indonesia selected for collecting field populations of *Ostrinia furnacalis* in 2013–2015 during the peak corn season in each province. With the exception of the Yogyakarta Special Region, these provinces were the top corn-producing areas in the country.

Egg masses, larvae, and pupae of *O. furnacalis* were collected from six provinces in Indonesia: three on Java Island (East Java, Central Java, and Yogyakarta Special Region), two on Sumatra Island (North Sumatra and Lampung), and the province of Gorontalo on Sulawesi (Figure 1). With the exception of Yogyakarta, these provinces are the major corn production sites in Indonesia, and insect pressure in these provinces were prominent based on the previous field observations. Field collections were made from non-*Bt* corn plants between 55 and 70 days after sowing with the intention of collecting insects from the second generation of *O. furnacalis*. Twenty-four populations were collected from 14 districts distributed in the six selected provinces between November 2013 and May 2015. The number of collected *O. furnacalis* from each location varied, and 83% of the collected populations were between 65 and 113 larvae and pupae. Only three of the collections were egg masses, each obtained from three different sites. The lowest number of *O. furnacalis* collected from one population was 12 larvae + 12 pupae + 1 egg mass. Each population was collected from at least two corn farms in one village to capture the genetic variability among individuals within a population and also because of the low infestations of *O. furnacalis* during the period of collection.

*O. furnacalis* at different life stages collected from the fields were handled using different methods. *O. furnacalis* egg masses were transferred into jars (18 cm tall, 7 cm diameter) layered with wetted filter paper to maintain the freshness of the eggs. Newly hatched larvae and bigger larvae were transferred individually into clear plastic containers with a screw cap (4.3 cm tall, 3.3 cm diameter) containing an artificial diet (ca. 5 g) [48] to minimize field-derived diseases. Collected pupae were put together in a container cup (the same size as for the larvae layered with filter paper) with a maximum of 10 pupae in each container. All collected larvae were healthy and pupated. Pupae produced from collected larvae, as well as those collected directly from the field, were placed into a Petri dish (9 cm diameter) layered with filter paper, and the Petri dish with the pupae was placed in a wire mesh cage (20 cm in diameter and 20 cm in height) upon arrival in the laboratory. Moisture was maintained by adding water to the filter paper in the plastic cups containing egg masses. *Ostrinia furnacalis* populations collected from different locations were reared in separate cups, trays, and cages to avoid contamination between populations. Additional details are provided below in “Insect-Rearing Procedure”.

### 5.2. Insect-Rearing Procedure

The field-collected and laboratory *O. furnacalis* populations were reared using a similar artificial diet and standardized laboratory procedures [48]. Larvae were fed on a red-bean-based artificial diet (2–3 larvae per ~5 g of diet) in clear plastic cups (3.3 cm in diameter and 4.3 cm height) until pupation. A maximum of fifty cups were placed in a plastic tray. The trays were labelled with the locations of the populations. Pupae were collected daily and approximately 200 pupae were placed in a Petri dish (9 cm in diameter). The Petri dish containing the pupae were placed in the middle of a mating cage made of wire (21 cm in diameter and 21 cm height) covered with white paper on the top for oviposition. Emerging adults were fed with a 10% honey solution, and wet cotton was placed in the cage for maintaining high relative humidity during the day. The paper containing egg masses was removed every other day or daily as needed to collect larvae of similar ages. Egg masses were incubated in glass jars (6.5 cm in diameter and 15.5 cm height) containing moistened filter paper until hatching. A portion of the newly hatched larvae (ca. 300 larvae) was transferred individually into plastic cups as described above for the next generations, and the other portion was used for bioassays. All insect life stages were incubated at room temperature (24–28 °C) with relative humidity ranges of 60–85%. All equipment for making the artificial diet as well as for rearing was semi-sterilized by dipping in 10% sodium hypochlorite (SC Johnson, Indonesia). If mortality occurred during mass rearing, dead larvae were removed immediately from the colony to prevent contamination during mass rearing.

### 5.3. Susceptibility of O. furnacalis to Cry1A.105 and Cry2Ab2

The proteins (Cry1A.105 and Cry2Ab2) were provided by Bayer CropScience, St. Louis, MO, USA. Bioassays were carried out using mostly F_2_ generation *O. furnacalis* neonates and a few using the F_1_ or F_3_ generation. The F_3_ generation was used when neonates of F_2_ was not sufficient to do the whole bioassays. The larvae of F_2_ and F_3_ were used if the F_1_ neonates in uniform age were inadequate for the conduction of the bioassay. Bioassays were carried out by following the diet-dipping procedure described by Trisyono et al. [49] by dipping the diet in protein solutions or distilled water. A cube of diet (1 cm × 1 cm × 1 cm) was dipped for 10 s in a treated or control solution and then air dried for 20 min. After drying, the diet was transferred into a plastic cup of similar size to the ones used for rearing. For each replication, ten newly hatched *O. furnacalis* larvae were transferred individually into two plastic cups containing the treated or control diet (5 larvae per cup). Each treatment was replicated 3–5 times. In the Cry1A.105 bioassays, 10 concentrations from 0.002 to 48 µg/mL Cry1A.105 (three-fold dilutions) were tested to determine LC_50_ and LC_95_ of 24 field-collected *O. furnacalis* populations and the laboratory population. For Cry2Ab2, 10 concentrations ranging from 0.0007 to 15.5 µg/mL (three-fold dilutions) and the control were used for all bioassays to determine LC_50_ and LC_95_ against the same F_1_–F_3_ of field-collected and laboratory populations. Based on the results from the preliminary bioassays, the concentrations used for Cry1A.105 and Cry2Ab2 were different due to varying response of *O. furnacalis* to each protein. The selected concentrations based on the preliminary test were expected to result in larval mortality ranging from 2% to 98%. Prior to the actual assays, preliminary tests of both proteins were carried out using 10 newly hatched larvae per concentration in three replicates. Larvae were exposed to the treated or control diet continuously, and observed mortality was recorded 7 days after they were placed in the plastic cups containing the artificial diet.

### 5.4. Data Analysis

Probit analysis [50] was carried out using the PoloJR program within PoloSuite, Version 2.1 (LeOra Software 2016) to determine LC_50_ and LC_95_ values and their 95% fiducial limits (FLs) for each protein and population. The relative resistance ratios (RR) were calculated by dividing the values of LC_50_ of field populations by that of the laboratory population [51]. The LC_95_ and LC_99_ and their lower and upper limits for the Cry1A.105 and Cry2Ab2 proteins were determined from pooled baseline concentration–mortality data (24 field-collected populations) using the program PoloJr, and the estimated LC_99_ and its lower and upper limits of 95% were proposed as candidate diagnostic concentrations for each protein, as they killed 99% of the susceptible population [52,53].

## Figures and Tables

**Table 1:** Susceptibility to Cry1A.105 of field-collected populations of *Ostrinia furnacalis* from major corn-growing areas in Indonesia.

Population			N	Slope (±SE)	LC_50_ (95% FL) (µg/mL)	LC_95_ (95% FL) (µg/mL)	RR ^a^	χ^2 c^
Province	District	Village						
North Sumatra	Langkat	Pasar VI Kwala Mencirim	440	2.05 ± 0.32	0.035 (0.012–0.066)	0.224 (0.112–1.173)	0.5	5.90
		Purwo Binangun	330	1.44 ± 0.32	0.039 (0.001–108)	0.535 (0.176–355.074)	0.6	6.38
	Deli Serdang	SM Diski	330	1.96 ± 0.42	0.010 (0.001–0.020)	0.069 (0.031–3.746)	0.1	1.31
		Suka Dame	330	1.27 ± 0.36	0.013 (N/A–N/A) *	0.250 (N/A–N/A) *	0.2	8.70 **
	Simalungun	Silenduk	550	0.77 ± 0.07	0.058 (0.031–0.105)	7.872 (2.647–45.801)	0.9	7.74
		Tangga Batu	550	0.93 ± 0.10	0.047 (0.023–0.081)	2.746 (1.311–8.273)	0.7	5.27
Lampung	South Lampung	Lematang	330	1.57 ± 0.42	0.045 (0.008–0.084)	0.495 (0.254–3.374)	0.7	3.57
		Kelau	550	1.33 ± 0.13	0.080 (0.044–0.132)	1.389 (0.701–4.166)	1.2	9.12
	Central Lampung	Trimurjo	330	0.86 ± 0.15	0.185 (0.066–0.357)	15.233 (5.345–127.917)	2.8	2.49
	Metro	Mulyojati	550	1.46 ± 0.20	0.032 (0.015–0.055)	0.429 (0.214–1.606)	0.5	4.17
	East Lampung	Gondang Rejo	550	1.70 ± 0.21	0.137 (0.102–0.176)	1.274 (0.906–2.003)	2.0	1.68
Gorontalo	Pohuwato	Manawa	550	1.65 ± 0.17	0.313 (0.238–0.403)	3.085 (2.062–5.408)	4.7	1.80
	Gorontalo	Tenilo	550	1.37 ± 0.20	0.273 (0.115–0.451)	4.316 (2.237–16.451)	4.1	2.18
Central Java	Purworejo	Ketawangrejo	550	1.38 ± 0.18	0.166 (0.071–0.292)	2.597 (1.148–16.906)	2.5	3.59
		Wonosari	550	1.06 ± 0.11	0.401 (0.233–0.630)	14.521 (7.038–45.194)	6.0	4.76
Yogyakarta	Sleman	Purwomartani	550	0.96 ± 0.11	0.051 (0.020–0.104)	2.604 (0.790–38.314)	0.8	6.87
		Widodomartani	550	1.02 ± 0.13	0.081 (0.022–0.197)	3.336 (0.932–98.888)	1.2	8.05
	Kulon Progo	Kedungsari	550	0.95 ± 0.10	0.053 (0.028–0.090)	2.907 (1.199–12.762)	0.8	5.77
		Kedundang	550	0.81 ± 0.07	0.101 (0.059–0.173)	0.730 (3.970–49.262)	1.5	6.55
East Java	Kediri	Papar	550	1.08 ± 0.10	0.043 (0.029–0.063)	1.404 (0.716–3.727)	0.6	3.90
		Mekikis	550	0.88 ± 0.11	0.006 (0.002–0.010)	0.418 (0.170–2.088)	0.1	3.16
	Nganjuk	Watu Dandang	550	0.77 ± 0.09	0.014 (0.006–0.025)	1.905 (0.659–12.081)	0.2	5.24
		Banjar Sari	550	0.83 ± 0.11	0.009 (0.003–0.020)	0.858 (0.231–20.417)	0.1	6.38
		Malangsari	550	0.91 ± 0.11	0.010 (0.004–0.021)	0.664 (0.225–6.057)	0.2	4.84
Laboratory ^b^			330	1.47 ± 0.30	0.067 (0.004–0.164)	0.890 (0.336–49.547)	1.0	6.11

N = number of larvae. SE = standard error. 95% FL = 95% fiducial limits. ^a^ Resistance ratios (RRs) were calculated by dividing the values of LC_50_ of field populations by that of the laboratory population. ^b^ Collected from Yogyakarta in 2009 and maintained in the laboratory without *Bt* or insecticide selection. * The program did not give the 95% FL values. ^c^ Double asterisk (**) indicates significant differences based on the *p*-value (*α* = 0.05).

**Table 2:** Susceptibility to Cry2Ab2 of field-collected populations of *Ostrinia furnacalis* from major corn-growing areas in Indonesia.

Population			N	Slope (±SE)	LC_50_ (95% FL) (µg/mL)	LC_95_ (95% FL) (µg/mL)	RR ^a^	χ^2 c^
Province	District	Village						
North Sumatra	Langkat	Pasar VI Kwala Mencirim	440	1.18 ± 0.16	1.527 (0.657–3.478)	37.665 (11.088–1150.551)	0.7	4.12
		Purwo Binangun	440	0.91 ± 0.19	4.361 (1.886–10.182)	277.584 (62.854–14,840.756)	1.9	2.70
	Deli Serdang	SM Diski	330	0.96 ± 0.18	3.416 (1.311–17.291)	180.121 (27.658–426,921.2)	1.5	3.44
		Suka Dame	440	2.23 ± 0.50	2.054 (0.490–3.540)	11.185 (5.908–130.605)	0.9	4.57
	Simalungun	Silenduk	550	0.83 ± 0.10	1.062 (0.673–1.684)	99.658 (36.621–502.575)	0.5	2.18
		Tangga Batu	440	1.58 ± 0.39	4.490 (2.313–7.002)	49.531 (22.220–511.071)	2.0	2.24
Lampung	South Lampung	Lematang	550	1.19 ± 0.23	0.667 (0.268–1.181)	16.275 (6.610–132.775)	0.3	4.03
		Kelau	330	2.17 ± 0.56	4.018 (1.242–6.506)	23.015 (11.971–370.390)	1.8	5.09
	Central Lampung	Trimurjo	550	1.09 ± 0.19	2.074 (0.396–5.564)	67.414 (17.091–12,346.001)	0.9	3.89
	Metro	Mulyojati	550	0.99 ± 0.20	4.034 (0.966–12.182)	183.278 (36.318–284,609.920)	1.8	5.50
	East Lampung	Gondang Rejo	330	1.22 ± 0.27	1.662 (0.720–2.884)	37.164 (16.372–205.583)	0.7	1.71
Gorontalo	Pohuwato	Manawa	330	1.17 ± 0.28	1.590 (0.040–4.273)	40.540 (10.897–459,863.240)	0.7	5.71
	Gorontalo	Tenilo	550	1.01 ± 0.11	0.525 (0.212–1.110)	22.666 (6.744–387.286)	0.2	7.83
Central Java	Purworejo	Ketawangrejo	550	1.40 ± 0.16	0.989 (0.481–1.824)	14.861 (5.979–122.810)	0.4	4.39
		Wonosari	550	1.08 ± 0.11	0.254 (0.177–0.351)	8.371 (4.952–16.923)	0.1	2.20
Yogyakarta	Sleman	Purwomartani	550	1.09 ± 0.11	0.322 (0.175–0.550)	10.463 (4.533–41.801)	0.1	6.49
		Widodomartani	550	1.26 ± 0.16	0.163 (0.077–0.272)	3.320 (1.520–16.269)	0.1	2.57
	Kulon Progo	Kedungsari	550	1.20 ± 0.12	0.741 (0.401–1.340)	17.198 (6.732–104.303)	0.3	6.27
		Kedundang	550	1.01 ± 0.12	0.276 (0.134–0.477)	11.710 (4.766–61.882)	0.1	4.18
East Java	Kediri	Papar	330	0.66 ± 0.07	0.101 (0.058–0.176)	30.870 (10.553–144.161)	0.1	5.41
		Mekikis	330	0.62 ± 0.07	0.095 (0.039–0.229)	41.121 (8.410–667.267)	0.1	11.65
	Nganjuk	Watu Dandang	550	1.01 ± 0.10	0.585 (0.315–1.102)	24.771 (8.645–164.730)	0.3	8.48
		Banjar Sari	550	0.61 ± 0.06	0.044 (0.019–0.099)	22.509 (4.654–375.668)	0.1	12.86
		Malangsari	550	0.97 ± 0.08	0.522 (0.309–0.907)	25.653 (9.980–113.189)	0.2	8.43
Laboratory ^b^			440	1.64 ± 0.48	2.276 (0.916–3.803)	22.984 (12.650–77.799)	1.0	0.62

N = number of larvae. SE = standard error. 95% FL = 95% fiducial limits. ^a^ Resistance ratios (RRs) were calculated by dividing the values of LC_50_ of field populations by that of the laboratory population. ^b^ Collected from Yogyakarta in 2009 and maintained in the laboratory without *Bt* or insecticide selection. ^c^ No significant differences based on the *p*-value (α = 0.05).

**Table 3:** Candidate diagnostic concentrations of Cry1A.105 and Cry2Ab2 estimated using baseline susceptibility data of 24 field-collected populations of *Ostrinia furnacalis*.

Protein	Slope	No. Larvae	LC_95_ (95% FL) (µg/mL)	LC_99_ (95% FL) (µg/mL)
Cry1A.105	0.99 ± 0.03	11,200	2.720 (1.680–5.158) a	13.240 (6.716–33.831) b
Cry2Ab2	1.04 ± 0.03	11,440	28.050 (13.600–89.795) a	127.320 (46.616–676.792) a

SE = standard error. 95% FL = 95% fiducial limits. For each protein, LC_95_ and LC_99_ values followed by different letters were significantly different based on non-overlapping 95% fiducial limits.

## Data Availability

Not applicable.

## References

[1] Gerpacio R.V., Pingali P.L. (2007). Tropical and Subtropical Maize in Asia: Production Systems, Constraints, and Research Priorities.

[2] Areekul S., Skulpanich U., Teeravate P. (1964). Some studies on the control of corn borer in Thailand. Agric. Nat. Resour..

[3] Camarao G.C. (1976). Population dynamics of the cornborer, *Ostrinia furnacalis* (Guenee), I. Life cycle, behavior, and generation cycles. Philipp. Entomol..

[4] Nafus D.M., Schreiner I.H. (1987). Location of *Ostrinia furnacalis* (Lepidoptera: Pyralidae) eggs and larvae on sweet corn in relation to plant growth stage. J. Econ. Entomol..

[5] Nafus D.M., Schreiner I.H. (1991). Review of the biology and control of the Asian corn borer, *Ostrinia furnacalis* (Lep: Pyralidae). Trop. Pest Manag..

[6] Da-Lopez Y.F., Trisyono Y.A., Witjaksono W., Subiadi S. (2014). Distribution pattern of *Ostrinia furnacalis* Guenée (Lepidoptera Crambidae) egg-mass on maize-field. J. Entomol. Indones..

[7] Trisyono Y.A., Aryuwandari V.E.F., Andika I.P., Sinulingga N.G.H. (2020). Assessment on the Economic Importance of Corn Borers in Indonesia.

[8] Morallo-Rejesus B., Buctuanon E.M., Rejesus R.S. (1990). Defining the economic threshold determinants for the Asian corn borer, *Ostrinia furnacalis* (Guenee) in the Philippines. Int. J. Pest Manag..

[9] Subiadi S., Trisyono Y.A., Martono E. (2014). Economic injury level (EIL) of *Ostrinia furnacalis* (Lepidoptera: Crambidae) larvae on three growth stages of corn. J. Entomol. Indones..

[10] CABI *Ostrinia furnacalis* (Asian Corn Borer). In: Invasive Species Compendium. https://www.cabi.org/isc/datasheet/38026.

[11] Plantwise Knowledge Bank Asian Corn Borer (*Ostrinia furnacalis*). https://www.plantwise.org/knowledgebank/datasheet/38026.

[12] Hussein M.Y., Kameldeer A.K. (1988). A field study on the oviposition of *Ostrinia furnacalis* Guenee (Lepidoptera: Pyralidae) on maize in Selangor, Malaysia. Int. J. Pest Manag..

[13] Brookes G., Dinh T.X. (2021). The impact of using Genetically Modified (GM) corn/maize in Vietnam: Results of the first farm-level survey. GM Crops Food.

[14] International Service for the Acquisition of Agri-Biotech Applications (ISAAA) (2019). Global Status of Commercialized Biotech/GM Crops in 2019: Biotech Crops Drive Socio-Economic Development and Sustainable Environment in the New Frontier.

[15] ISAAA ISAAA’s GM Approval Database. http://www.isaaa.org/gmapprovaldatabase/.

[16] Biosafety Clearing-House (BCH) Country’s Decisions and Other Communications. https://bch.cbd.int/en/registries/living-modified-organisms.

[17] Indonesia Biosafety Clearing House Keputusan Aman-Pangan. https://indonesiabch.menlhk.go.id/surat-keputusan/.

[18] Erasmus A., Marais J., Van den Berg J. (2016). Movement and survival of Busseola fusca (Lepidoptera: Noctuidae) larvae within maize plantings with different ratios of non-Bt and Bt seed. Pest. Manag. Sci..

[19] Botha A.S., Erasmus A., du Plessis H., Van den Berg J. (2019). Efficacy of Bt maize for control of Spodoptera frugiperda (Lepidoptera: Noctuidae) in South Africa. J. Econ. Entomol..

[20] Alcantara E., Atienza M.M., Camacho L., Parimi S. (2021). Baseline susceptibility of Philippine *Ostrinia furnacalis* (Lepidoptera: Crambidae) populations to insecticidal Cry1A. 105 and Cry2Ab2 proteins and validation of candidate diagnostic concentration for monitoring resistance. Biodiversitass.

[21] James C. (2011). Global Status of Commercialized Biotech/GM Crops: 2011.

[22] International Service for the Acquisition of Agri-Biotech Applications (ISAAA) (2016). Global Status of Commercialized Biotech/GM Crops: 2016.

[23] Department of Agriculture List of Registered Plant-Incorporated Protectants Derived from Modern Biotechnology 2021. Department of Agriculture Quezon City, Philippines. https://fpa.da.gov.ph/NW/images/FPAfiles/DATA/Regulation/Pesticide/Files-2021/ListofRegisteredPIP12312021.pdf.

[24] Gould F. (1998). Sustainability of transgenic insecticidal cultivars: Integrating pest genetics and ecology. Annu. Rev. Entomol..

[25] Tabashnik B.E. (2008). Delaying insect resistance to transgenic crops. Proc. Natl. Acad. Sci. USA.

[26] Tabashnik B.E., Carrière Y. (2020). Evaluating cross-resistance between Vip and Cry toxins of Bacillus thuringiensis. J. Econ. Entomol..

[27] Roush R.T. (1998). Two–toxin strategies for management of insecticidal transgenic crops: Can pyramiding succeed where pesticide mixtures have not?. Phil. Trans. R. Soc. Lond. B.

[28] Zhao J.-Z., Cao J., Li Y., Collins H.L., Roush R.T., Earle E.D., Shelton A.M. (2003). Transgenic plants expressing two Bacillus thuringiensis toxins delay insect resistance evolution. Nat. Biotechnol..

[29] Carrière Y., Crickmore N., Tabashnik B.E. (2015). Optimizing pyramided transgenic Bt crops for sustainable pest management. Nat. Biotechnol..

[30] Blanco C.A., Storer N.P., Abel C.A., Jackson R., Leonard R., Lopez J.D., Payne G., Siegfried B.D., Spencer T., N-Vargas A.P.T. (2008). Baseline susceptibility of tobacco budworm (Lepidoptera: Noctuidae) to Cry1F toxin from Bacillus thuringiensis. J. Econ. Entomol..

[31] Leite N.A., Pereira R.M., Durigan M.R., Amado D., Fatoretto J., Medeiros F.C.L., Omoto C. (2018). Susceptibility of Brazilian populations of Helicoverpa armigera and Helicoverpa zea (Lepidoptera: Noctuidae) to Vip3Aa20. J. Econ. Entomol..

[32] Robertson J.L., Preisler H.K. (1992). Pesticide Bioassays with Arthropods.

[33] Liu X., Liu S., Long Y., Wang Y., Zhao W., Shwe S.M., Wang Z., He K., Bai S. (2022). Baseline susceptibility and resistance allele frequency in *Ostrinia furnacalis* in relation to Cry1Ab toxins in China. Toxins.

[34] Tabashnik B.E., Re Y.C., Dennehy T.J., Morin S., Sisterson M.S., Roush R.T., Shelton A.M., Zhao J.-Z. (2003). Insect resistance to transgenic Bt crops: Lessons from the laboratory and field. J. Econ. Entomol..

[35] Huang F., Andow D.A., Buschman L.L. (2011). Success of the high-dose/refuge resistance management strategy after 15 years of Bt crop use in North America: Bt crops and resistance management. Entomol. Exp. Appl..

[36] Alcantara E., Estrada A., Alpuerto V., Head G. (2011). Monitoring Cry1Ab Susceptibility in Asian corn borer (Lepidoptera: Crambidae) on Bt corn in the Philippines. Crop Prot..

[37] Marçon P.C.R.G., Young L.J., Steffey K.L., Siegfried B.D. (1999). Baseline susceptibility of European corn borer (Lepidoptera: Crambidae) to Bacillus thuringiensis toxins. J. Econ. Entomol..

[38] Jalali S.K., Yadavalli L., Ojha R., Kumar P., Sulaikhabeevi S.B., Sharma R., Nair R., Kadanur R.C., Kamath S.P., Komarlingam M.S. (2015). Baseline sensitivity of maize borers in India to the Bacillus thuringiensis insecticidal proteins Cry1A.105 and Cry2Ab2: Bt baseline sensitivity of Indian maize Lepidopteran Pests. Pest. Manag. Sci..

[39] Le D.K., Le Q.K., Tran T.T.H., Nguyen D.V., Dao T.H., Nguyen T.T., Truong X.L., Nguyen Q.C., Pham H.P., Phan T.T.T. (2019). Baseline susceptibility of Asian corn borer (*Ostrinia furnacalis* (Guenée)) populations in Vietnam to Cry1Ab insecticidal protein. J. Asia-Pac. Entomol..

[40] He K., Wang Z., Wen L., Bai S., Ma X., Yao Z. (2005). Determination of baseline susceptibility to Cry1Ab protein for Asian corn borer (Lep., Crambidae). J. Appl. Entomol..

[41] Li G., Huang J., Ji T., Tian C., Zhao X., Feng H. (2020). Baseline susceptibility and resistance allele frequency in *Ostrinia furnacalis* related to Cry1 toxins in the Huanghuaihai summer corn region of China. Pest Manag. Sci..

[42] Kranthi K.R., Kranthi S., Wanjari R.R. (2001). Baseline toxicity of Cry1A toxins to Helicoverpa armigera (Hubner) (Lepidoptera: Noctuidae) in India. Int. J. Pest Manag..

[43] Bird L.J., Akhurst R.J. (2007). Variation in susceptibility of Helicoverpa armigera (Hübner) and Helicoverpa punctigera (Wallengren) (Lepidoptera: Noctuidae) in Australia to two Bacillus thuringiensis Toxins. J. Invertebr. Pathol..

[44] Siegfried B.D., Spencer T., Crespo A.L., Storer N.P., Head G.P., Owens E.D., Guyer D. (2007). Ten years of Bt resistance monitoring in the European corn borer: What we know, what we don’t know, and what we can do better. Am. Entomol..

[45] Hernández-Rodríguez C.S., Hernández-Martínez P., Van Rie J., Escriche B., Ferré J. (2013). Shared midgut binding sites for Cry1A.105, Cry1Aa, Cry1Ab, Cry1Ac and Cry1Fa proteins from Bacillus thuringiensis in two important corn pests, Ostrinia nubilalis and Spodoptera frugiperda. PLoS ONE.

[46] Marçon P.C.R.G., Siegfried B.D., Spencer T., Hutchison W.D. (2000). Development of diagnostic concentrations for monitoring Bacillus thuringiensis resistance in European corn borer (Lepidoptera: Crambidae). J. Econ. Entomol..

[47] Monsanto Company Petition for the Determination of Non-Regulated Status for MON 89034. https://www.aphis.usda.gov/brs/aphisdocs/06_29801p.pdf.

[48] Rahayu T., Trisyono Y.A., Witjaksono (2018). Fitness of Asian corn borer, *Ostrinia furnacalis* (Lepidoptera: Crambidae) reared in an artificial diet. J. Asia-Pac. Entomol..

[49] Trisyono Y.A., Rahayu S.T.S., Margino S. (2004). Bioactivity of a Bacillus thuringiensis Cry1Ac toxin to Spodoptera litura. J. Perlindungan Tanam. Indones..

[50] Finney D.J. (1971). Probit Analysis.

[51] Oppenoorth F.J., Welling W., Wilkinson C.F. (1976). Biochemistry and Physiology of Resistance. Insecticide Biochemistry and Physiology.

[52] Roush R.T., Miller G.L. (1986). Considerations for design of insecticide resistance monitoring programs. J. Econ. Entomol..

[53] Menger J., Beauzay P., Chirumamilla A., Dierks C., Gavloski J., Glogoza P., Hamilton K., Hodgson E.W., Knodel J.J., MacRae I.V. (2020). Implementation of a diagnostic-concentration bioassay for detection of susceptibility to pyrethroids in soybean aphid (Hemiptera: Aphididae). J. Econ. Entomol..

